# Lymphedema of the Arm after COVID-19 Vaccination in a Patient with Hidden Breast Cancer and Paraneoplastic Dermatomyositis

**DOI:** 10.3390/vaccines10081219

**Published:** 2022-07-30

**Authors:** Cristina Aimo, Elena Biancamaria Mariotti, Alberto Corrà, Lavinia Quintarelli, Beatrice Bianchi, Alice Verdelli, Valentina Ruffo di Calabria, Marzia Caproni

**Affiliations:** 1 Section of Dermatology, Department of Health Sciences, University of Florence, 50125 Florence, Italy; elenabiancamaria.mariotti@unifi.it (E.B.M.); alberto.corra@unifi.it (A.C.); lavinia.quintarelli@unifi.it (L.Q.); beatrice.bianchi@unifi.it (B.B.); alice.verdelli@unifi.it (A.V.); valentina.ruffodicalabria@unifi.it (V.R.d.C.); marzia.caproni@unifi.it (M.C.); 2 Rare Diseases Unit, Azienda USL Toscana Centro, Section of Dermatology, Department of Health Sciences, University of Florence, European Reference Network Skin Member, 50125 Florence, Italy

**Keywords:** COVID-19 vaccine, adverse reaction, lymphedema

## Abstract

The pandemic outbreak of Coronavirus Disease 2019 (COVID-19) led to the development of mRNA vaccines. With the extensive vaccination campaign performed worldwide, many adverse reactions to these drugs have been reported in the literature. Although most of them are mild and self-limiting, they may sometimes cause psychological stress and require efforts to make a differential diagnosis with other conditions. This is the case of lymphadenopathies and lymphedema in patients with a history of cancer. Herein we present a case of lymphedema of the arm developed ten days after a VAXZEVRIA COVID-19 vaccine shot in a patient who had concomitant signs and symptoms compatible with a diagnosis of dermatomyositis. It was later classified as paraneoplastic as instrumental investigation revealed a breast carcinoma contralateral to the site of vaccine injection. With this report we ponder an adverse reaction to COVID-19 vaccination with the aim of bringing new data for clinicians who face similar clinical presentations, particularly controversial for radiologists and oncologists.

## 1. Introduction

The coronavirus disease 2019 (COVID-19) pandemic outbreak led to research, development, and use of messenger RNA (mRNA) vaccines. Among these are listed Pfizer-BioNTech, Moderna, Janssen–Johnson & Johnson, Vaxzevria (previously COVID-19 Vaccine AstraZeneca) and Sputnik-V. The most widely used vaccines worldwide have been Pfizer-BioNTech and Moderna. Concerning the Vaxzevria vaccine, which is given as a set of two injections separated in time by at least 10 weeks [1], some countries have suspended its use or limited it to elderly people at higher risk for severe COVID-19 illness due to concerns over the very rare side effects of the vaccine in younger individuals. Along with these issues, many other concerns over the safety have been raised since the approval of COVID-19 vaccines [2,3,4]. It has been a topic of discussion, for example, whether patients with central nervous system (CNS) demyelinating diseases should undergo vaccination, as limited information is available on associations between COVID-19 vaccines and CNS diseases.

However, as with other viral vaccines, the risk of COVID-19 vaccine-associated CNS demyelinating disease was found to be low [5,6]. Immunosuppressive and immunomodulating therapies while receiving COVID-19 vaccination have been also object of discussion. The data available so far encourage vaccination in most situations [7,8,9,10]. The same conclusions have been reached for cancer patients, even when already in treatment. In fact, it has been stated that patients with breast or gynecological cancers who are receiving treatment or are in the five-year post treatment period should be included in the priority group for severe acute respiratory syndrome coronavirus-2 (SARS-CoV-2) vaccination [11].

Nowadays, despite proven efficacy and safety profiles of COVID-19 vaccines, there is still a substantial number of people who express vaccine hesitancy. This phenomenon may be amplified by misinformation [12]. Accordingly, it is important to continue collecting data concerning safety and adverse effects.

The most common reported side effects for the COVID-19 vaccine are similar to those reported for non mRNA vaccines. They include pain in the injection site, fatigue, headaches, fever, chills, muscle, and joint pains and are associated with the desired immune activation mediated by vaccines. Severe allergic reactions, e.g., anaphylaxis, are reported to occur very rarely (9.9–28.4 cases per 1 million doses) [13]. Thrombosis with thrombocytopenia syndrome (TTS) was confirmed in 17 cases out of more than 8 million doses of Janssen–Johnson & Johnson vaccine, all occurring in women aged 18–59 years (median age 37 years). Although rare, thromboembolic events with concurrent thrombocytopenia events have been also reported following administration of mRNA COVID-19 vaccines. There are a small number (0.8 per million doses of BNT162b2 or mRNA-1273) of immune thrombocytopenia reports that did not modify its overall incidence. Moreover, reports have suggested that there is an association between COVID-19 vaccination and myocarditis and pericarditis. However, given their low frequency, the CDC determined that the benefits of vaccination outweighed the risks [13]. Lymphadenopathy, among the other side effects, has also been reported [14].

Adenopathy related to recent vaccination status is not an uncommon benign etiology as it has been documented following smallpox, Bacillus Calmette–Guerin (BCG), anthrax, and Human Papilloma Virus (HPV) [15] vaccinations. It may be interpreted as a delayed hypersensitivity reaction to SARS-CoV-2 vaccination displaying a localized inflammatory response: immune cells of the lymph nodes nearby the vaccination site may proliferate as they become exposed to the vaccine antigen causing lymphadenopathy and reducing lymph nodal drainage. Regarding the outcome of the lymphadenopathy post-COVID-19 vaccination, some studies reported a maximum duration of 10 days [16], however there are some case reports of durations of up to 32 days [17].

Herein is reported the case of a patient with severe lymphedema of the left arm occurring after COVID-19 vaccination. While it initially raised the suspicion of a hidden left breast cancer, it led instead to the diagnosis of a right breast cancer lymphadenopathy and lymphedema involving the supraclavicular, infraclavicular, and axillary regions on the same side as the injection site, are mentioned among the possible side effects of COVID-19 vaccine but have been rarely reported before [17,18]. Differential diagnosis of unilateral axillary adenopathy is broad, including benign conditions such as inflammation, infection or trauma of the breast, thoracic wall, or arm but also malignancy, particularly breast cancer, head and neck cancers, lymphoma, and melanoma of the back and upper extremities, which have a predilection to metastasize to these lymph node stations.

Recognizing this association is crucial in patients with cancer, as failure to do so can lead to under- or over-diagnosis and under- or over-treatment as well as heightened anxiety. It has been reported, however, that it might not be easy to differentiate between benign and malignant nodal involvement especially when the vaccine is administrated on the same side as the tumor’s expected nodal drainage. In fact, benign metabolic hyperactivity and transient fluorodeoxyglucose (FDG) uptake in the lymph nodes of patients receiving positron emission tomography (PET) or CT scans after COVID-19 vaccination have been reported [19]. Therefore, accurate anamnesis is fundamental to assess the correct management.

## 2. Detailed Case Description

A 45-year-old woman with a history of alcohol and drug abuse presented at our dermatological service complaining of muscular and cutaneous manifestations. In anamnesis she presented hypercholesterolemia, hepatic steatosis, hiatal hernia, and anxious–depressive syndrome.

**Figure 1:**
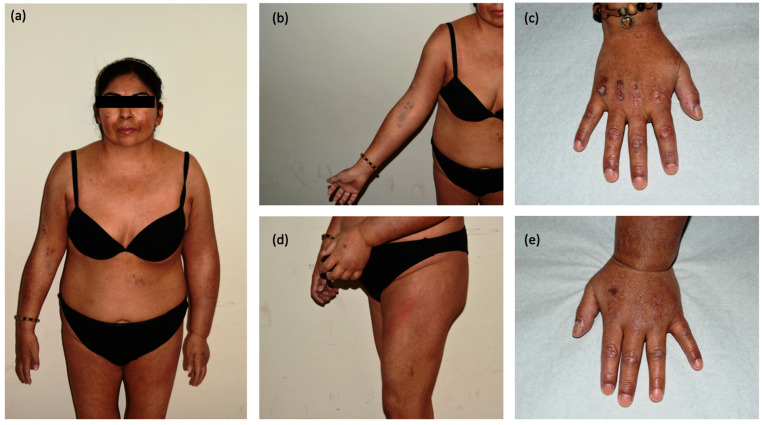
Clinical pictures of the patient at first medical contact: (**a**) full body photograph showing asymmetric edema mainly involving the left arm; (**b**) cutaneous ulcers over the right arm; (**c**) detail of the right hand showing Gottron’s papules and dystrophic cuticles; (**d**) Holster’s sign of the left tight; (**e**) detail of the left hand showing conspicuous edema.

She referred asthenia, dysphagia, and progressive proximal muscle weakness. Muscle weakness was not worsened by activity or improved by rest and did notget worse in the evening hours. At physical examination a dusky red rash was appreciable on the trunk, thighs, and periorbital regions. Violaceous papules were identifiable on the metacarpophalangeal and interphalangeal joints, elbows, and knees. On her hands, marked hyperkeratosis with fingertip crusts and periungual telangiectasias could also be observed. Along with these manifestations, her left arm, from the axillary region to the hand, was conspicuously edematous and non-painful (Figure 1).

Considering muscular and cutaneous manifestations, dermatomyositis was suspected [20]. A cutaneous biopsy was conducted. The histologic report although not specific, showing iper-orthokeratosis, epidermal atrophy, and homogenized collagen fibers, did not exclude the clinical suspicions [21]. The diagnosis was then confirmed by elevation of muscle enzymes (CPK 1062 U/L, LDH 577 U/L, ALT 65 U/L, AST 103 U/L, Aldolase 4.2 UI/L) and an electromyography showing a myopathic pattern with activity compatible with myositis. Moreover, myositis-specific immunoblotting showed positivity for transcription intermediary factor 1-gamma (TIF-1-gamma) antibodies. Dysphagia was investigated with esophageal-duodenal endoscopy which documented a serrated stenosis of the superior esophageal sphincter and atony of the inferior esophageal tract, compatible with a myositis. As it is known that TIF-1γ-positive dermatomyositis has a strong association with malignancies [22], trans-vaginal ultrasound, total-body CT scan and abdominal ultrasound were requested and revealed a nodule in the right breast. For further investigation, bilateral mammography was performed, with the confirmation of a nodule of 23 mm in the right breast. A biopsy of the lesion was performed for histological evaluation, with a diagnosis of breast adenocarcinoma non breast cancer 1 (BRCA1) or breast cancer 2 (BRCA2) mutated. Since the edema of the left limb was suggestive of a secondary defect in lymphatic drainage, bilateral axillary sonography was performed with the findings of a reactive lymphadenopathy in the left axilla and of a pathologic lymph node in the right axilla. This finding excluded a linkage between left arm edema and breast carcinoma. Notably, when asked, the patient reported that ten days before she had Vaxzevria COVID-19 vaccine in the same arm.

**Figure 2:**
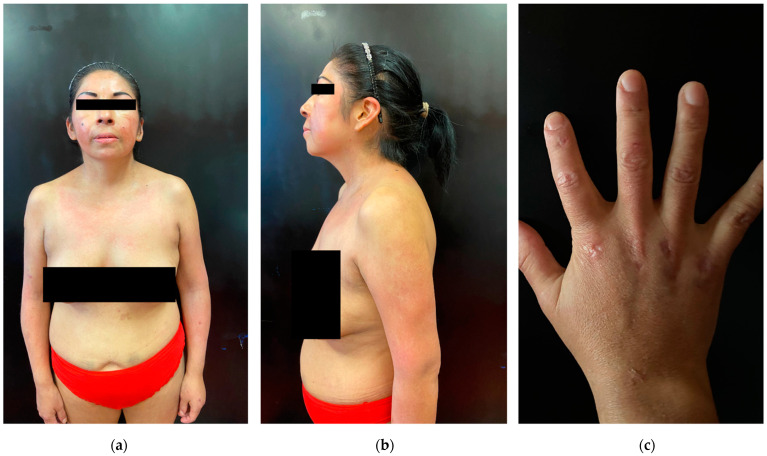
Clinical pictures of the patient after three weeks of therapy (**a**) marked improvement of edema and skin appearance with persistence of V-sign; (**b**) Gottron’s sign; (**c**) reduction of the left-hand edema with persistence of Gottron’s papules.

Waiting for surgical excision of the tumor, the patient was treated with intravenous corticosteroids (1 mg/kg/die) and immunoglobulins (25 g/die for 5 days) with partial improvement and reduction of muscular enzymes in three weeks. (Figure 2).

However, when the patient was admitted to the hospital for the scheduled surgery, she was in poor general condition. Marked edema of the periorbital region and of the inferior limbs configured nearly an anasarca. Notably, the left arm lymphedema had disappeared. The patient complained of an intense asthenia and dysphagia. She claimed to have stopped the steroid treatment by her own will. A pre-operative Positron Emission Tomography and Computed Tomography (PET-CT) scan confirmed the hypermetabolism of the right breast nodule, in the right axilla, and a diffuse and moderate hypermetabolism of the muscles, especially of the limbs, the neck, and the pectorals. The clinical and instrumental findings were suggestive of an exacerbation of dermatomyositis. Therefore, steroids were re-introduced along with a new cycle of intravenous immunoglobulins, with progressive improvement. When the patient’s conditions were considered permissive, she underwent surgery for the breast carcinoma. A right mastectomy was performed with concomitant sentinel lymph node biopsy (SNLB), result negative. In the days that followed surgery, it was complicated by a conspicuous hematoma of the site causing a rapid progressive hemorrhagic anemia with a beginning of hypovolemic shock. The patient underwent an urgent revision of the surgical site with an accurate hemostasis along with blood transfusions. Moreover, during the hospitalization, the patient developed high fever. An X-ray exam led to a diagnosis of pneumonia, most likely aspiration pneumonia. Despite antibiotic treatment, the rapid decrease of saturation forced the intubation. A subsequent otorhinolaryngology evaluation reported an intense swelling of the base of the tongue and edema of the epiglottis, restricting the airways by nearly 70%, with indication for a tracheostomy.

During the hospitalization, thirty days from the previous administration, a new cycle of endovenous immunoglobulin was administered. The patient, in good clinical conditions, was then discharged from the hospital with indications for a new administration of intravenous immunoglobulins thirty days apart from the previous ones. From an oncologic point of view, adjuvant chemotherapy was suggested.

## 3. Discussion

In the case reported, an adverse reaction to COVID-19 vaccination consisting of ipsilateral lymphadenopathy, led to the diagnosis of a breast cancer in a patient with concomitant paraneoplastic dermatomyositis. Dermatomyositis (DM) is listed among idiopathic inflammatory myopathies, a heterogeneous group of rare systemic autoimmune diseases primarily characterized by muscle weakness, often accompanied by extra muscular involvement. Along with proximal muscle weakness, DM is characterized by dermatological manifestations classified as pathognomonic (heliotrope rash, Gottron’s sign, Gottron’s papules), characteristics (V and shawl sign) and compatible. Other signs and symptoms may include pitting edema, dysphagia secondary to bulbar muscle weakness and nasal regurgitation of liquids or aspiration pneumonia and dyspnea [23]. As our patient exhibited pathognomonic signs for dermatomyositis (Gottron’s sign, Gottron’s papules), the diagnosis of DM was made. Proximal weakness and bulbar muscles weakness are characteristics of myasthenia gravis too. However, muscle weakness was not worsened by activity and improved by rest and did not get worse in the evening hours. The patient never showed asymmetric ptosis or variable diplopia, present in most cases of myasthenia gravis [24]. DM is associated with an underlying malignancy in 6–60% of cases. In these cases, it is considered a paraneoplastic syndrome. Among the others, ovarian cancer, lung cancer, pancreatic cancer, stomach cancer, colorectal cancer and non-Hodgkin’s lymphoma are reported to be the most frequently involved. The risk of malignancy is highest in patients aged 45–74 years at the time of diagnosis [25]. The association between DM and malignancies is particularly strong in those patients showing autoantibodies to TIF1γ or nuclear matrix protein 2 (NXP2). Moreover, anti-TIF1γ serum titer is correlated with myositis activity and with the presence of metastatic or recurrent malignancy [26]. In our case a diagnosis of paraneoplastic dermatomyositis was made since it was associated with a breast cancer. Although in the literature a few cases of dermatomyositis with TIF-1 gamma antibodies temporally related with SARS-CoV-2 vaccination [27,28] have been reported, retrospective and epidemiological studies failed to ascertain an association between DM and vaccines. In fact, no significant increase in the incidence of DM was reported after large vaccination campaigns [29]. The frequency of ipsilateral axillary lymphadenopathy is reported to be around 10% in the literature, similarly to data reported for patients in clinical trials attesting it to 14% [30]. A few cases from the literature describing this adverse effect have been summarized in Table 1.

The cases reported mainly involved women with a history of breast cancer, undergoing routinary imaging during follow-up. Lymphadenopathy was ipsilateral to the arm of the injection and contralateral to cancer side in every case except one (case 8). Latencybetween vaccination and lymph nodes enlargement was reported to be of 2–4 weeks. One case, involving a young woman with no history of cancer, was characterized for a long latency (four months) between the vaccine injection and the development of ipsilateral lymphadenopathy. However, an extensive diagnostic process concluded for an adverse reaction to vaccination. In most of these cases a biopsy was needed to ascertain the nature of the lymphadenopathy. This was mainly due to patients’ desire to put an end to the psychological stress they felt. Thus, while in the cases reported in the literature, lymphadenopathy led to misdiagnosis, unnecessary instrumental exams, and psychological stress, in our case the lymphedema contributed to the finding of a hidden breast cancer, contralateral to that of the vaccine injection. Moreover, the management of lymphedema was conditioned by the concomitant autoimmune disorder. Immunomodulatory/suppressive treatment led to its resolution in three weeks. In our case, the lymphedema, involving the whole ipsilateral arm and the hand, appeared to be particularly severe, making us interrogate whether in patients with a predisposing genetic background, concomitant phenomena such as vaccination and cancer may have amplified the overall immune response, partly due to the vaccine antigens injected, partly due to the immunosurveillance response to carcinogenesis process, causing augmented adverse reactions to vaccination. In fact, it has been observed that ipsilateral lymph node axillary uptake was more common in immunocompetent patients [34] and that in different pathological settings, environmental, pathogen or cancer derived stimuli may influence and dysregulate the interactions between immune cells and antibodies [35]. Data reported by Bshesh et al., seem to suggest that this side effect may be more common in oncological patients [36] but further research is needed to confirm if an association exists and if an underlying malignancy may amplify the hypersensitivity reaction observed.

In conclusion, lymphadenopathies post COVID-19 vaccination could be the result of an active immune response to the COVID-19 vaccine and could exhibit alarming aspects, being severe, long lasting, and sometimes causing borderline findings both in radiology and pathology with prominent multiple nodal enlargements and atypical cytopathologic features. Moreover, it has been outlined that it may be difficult to differentiate them from lymph node metastasis due to similarities in imaging and in the pattern of uptake on PET-CT [31].

These aspects represent a challenge when a patient with a history of cancer with a tendency to involve axillary lymph nodes presents with isolated axillary lymphadenopathy after a COVID-19 vaccine injection. For this reason, it has been recommended that patients with breast cancer, axillary lymphoma, and malignancy of the upper limb should not be vaccinated in the arm ipsilateral to the tumor’s expected nodal drainage [36].

For unilateral lymphadenopathy incidentally detected on imaging, a short-term follow-up of 4–12 weeks after the second vaccine dose has been suggested. In fact, this is the expected time for regression of enlarged lymph nodes in patients with no history of malignancy. Otherwise, if lymphadenopathy persists, biopsy is recommended [37]. A recent work published in *The American Journal of Roentgenology* [38], suggests delaying or rescheduling the FDG PET/CT if there is no urgency, as radiologists and oncologists may encounter transient FDG uptake in normal or enlarged axillary, supraclavicular and cervical lymph nodes after ipsilateral deltoid vaccination.The authors suggest performing FDG PET/TC at least 14 days after COVID-19 vaccination in patients with cancer, with an ideal timing of 4–6 weeks after vaccination, as vaccine-related lymphadenopathy on PET/TC up to 4–6 weeks after administration has been observed. Collecting data regarding this adverse effect to vaccination may help avoid errors of staging, patient anxiety and unnecessary investigations [39].

## 4. Conclusions

Many researchers emphasized that most of the adverse effects to vaccination are mild and self-limiting. Given the safety and efficacy of COVID-19 vaccinations in preventing severe complications, especially in the elderly and vulnerable people, vaccination continues to be strongly recommended, almost for every category of patients. However, as mass vaccination to COVID-19 continues, more adverse effects to vaccination will be encountered, possibly adding to pre-existing comorbidities. The appearance of new, especially non-painful lymphadenopathy, may be alarming. Increasing understanding of this adverse effect to vaccine may help clinicians to avoid unnecessary diagnostic procedures.

## Figures and Tables

**Table 1:** Case reports of lymphadenopathy post COVID-19 vaccination from the literature: case 1–6 [31]; case 7 [32]; case 8 [33]. (At https://www.1mdpi.com/ethics10 (accessed on 25 July 2022). It is reported that permission is not required for “reconstruction of your own table with data already published elsewhere”).

Case Report	Clinical Presentation	Cancer	Vaccine	Management
**1** 61-year-old woman	Multiple, enlarged lymph nodes in left axillary region on imaging studies in a routine 5-year follow-up	Right breast cancer with ipsilateral axillary metastasis	I dose of Vaxzevria in the left arm 16 days before	Biopsy (benign hyperplasia)
**2** 75-year-old woman	Multiple enlarged, round, and coffee bean-shaped lymph nodes in left axillary region during imaging studies in a routine 2.5-year follow-up	Right breast cancer without axillary metastases	II dose of the Pfizer-BioNTech 14 days before	Biopsy (reactive hyperplasia)
**3** 71-year-old woman	Smooth and diffuse enlargement of left axillary level I lymph nodes during 8-year surveillance exams	Right breast cancer without axillary metastasis	I dose of Vaxzevria in the left arm 8 and 14 days prior to current CT and US evaluation, respectively	Biopsy (benign hyperplasia)
**4** 73-year-old woman	Several enlarged lymph nodes in level I of her right axilla with one lymph node showing round shape during 10-year surveillance	Left breast cancer without axillary metastasis	Booster dose Vaxzevria–Pfizer-BioNTech cross-inoculation 28 days before	4–12 weeks follow-up
**5** 62-year-old woman	Unilateral left axillary lymphadenopathy during 2.5-year follow-up	Right breast cancer with ipsilateral axillary metastases	I dose of Vaxzevria in the left arm 3 weeks before	4–12 weeks follow-up
**6** 61-year-old woman	Right axillary lymphadenopathy detected during 3-year follow-up	Left breast cancer with ipsilateral axillary metastasis	I dose of Vaxzevria in the right arm 19 days before	3-month follow-up
**7** 34-year-old woman	Left axillary lymphadenopathy	She denied a medical history of past malignant tumors	II dose of CoronaVac in the left arm 4 months before	Biopsy (reactive hyperplasia)
**8** Woman, unknown age	Worsening of lymphedema on the cancer side	Right breast cancer with lymph node dissection	I dose of COVID-19 vaccine in the left arm	Conservative treatment

## Data Availability

Not applicable.
